# The number of privately treated tuberculosis cases in India: an estimation from drug sales data

**DOI:** 10.1016/S1473-3099(16)30259-6

**Published:** 2016-11

**Authors:** Nimalan Arinaminpathy, Deepak Batra, Sunil Khaparde, Thongsuanmung Vualnam, Nilesh Maheshwari, Lokesh Sharma, Sreenivas A Nair, Puneet Dewan

**Affiliations:** a MRC Centre for Outbreak Analysis and Modelling, School of Public Health, Imperial College London, London, UK; b IMS Health, New Delhi, India; c Central TB Division, Government of India, New Delhi, India; d World Health Organization, India Country Office, New Delhi, India; e Bill & Melinda Gates Foundation, New Delhi, India

## Abstract

**Background:**

Understanding the amount of tuberculosis managed by the private sector in India is crucial to understanding the true burden of the disease in the country, and thus globally. In the absence of quality surveillance data on privately treated patients, commercial drug sales data offer an empirical foundation for disease burden estimation.

**Methods:**

We used a large, nationally representative commercial dataset on sales of 189 anti-tuberculosis products available in India to calculate the amount of anti-tuberculosis treatment in the private sector in 2013–14. We corrected estimates using validation studies that audited prescriptions against tuberculosis diagnosis, and estimated uncertainty using Monte Carlo simulation. To address implications for numbers of patients with tuberculosis, we explored varying assumptions for average duration of tuberculosis treatment and accuracy of private diagnosis.

**Findings:**

There were 17·793 million patient-months (95% credible interval 16·709 million to 19·841 million) of anti-tuberculosis treatment in the private sector in 2014, twice as many as the public sector. If 40–60% of private-sector tuberculosis diagnoses are correct, and if private-sector tuberculosis treatment lasts on average 2–6 months, this implies that 1·19–5·34 million tuberculosis cases were treated in the private sector in 2014 alone. The midpoint of these ranges yields an estimate of 2·2 million cases, two to three times higher than currently assumed.

**Interpretation:**

India's private sector is treating an enormous number of patients for tuberculosis, appreciably higher than has been previously recognised. Accordingly, there is a re-doubled need to address this burden and to strengthen surveillance. Tuberculosis burden estimates in India and worldwide require revision.

**Funding:**

Bill & Melinda Gates Foundation.

## Introduction

Tuberculosis is a major global public health challenge.^1^ In 2014, 6·3 million cases of tuberculosis worldwide were reported to WHO, with India accounting for over a quarter of these cases, the highest of any country.^1^ Although standardised tuberculosis treatment in India is delivered by the public sector through the Revised National TB Control Programme (RNTCP), early diagnosis and treatment are hampered by the presence of a vast and unregulated private health-care sector.^2^, ^3^, ^4^, ^5^ Poor diagnostic practices in this sector prolong tuberculosis transmission by delaying diagnosis,^3^, ^5^, ^6^ whereas a general lack of counselling and support of treatment adherence hampers successful, relapse-free cure.^4^ Moreover, most cases treated in the private sector are never notified to public health authorities.^7^

Estimating the numbers of patients being treated in the private sector is important for several reasons: it provides information about the performance of a public system in detecting tuberculosis cases, while also helping in planning for government intervention in the private sector.^8^ Overall, it is crucial to know the scale of the problem: the undetected burden that exists outside the public health system. However, with a lack of systematic data on the private sector, arriving at these estimates has proven difficult.^9^ Instead, alternative approaches—such as that used by WHO—draw from expert opinion on the proportion of cases that are detected by the public sector.

In this work, we present an alternative approach. We build on earlier, innovative work that addressed the private market for tuberculosis drugs using comprehensive data on the sales of these drugs in the private sector.^10^ In the present study, using corresponding data for 2013 and 2014, we explored systematically the implications for tuberculosis burden (numbers of patients) being managed by the private sector in India, and compared this burden directly against that managed by the public sector.

## Methods

### Overview

We drew from a large, nationally representative dataset for private sector drug sales across the country, collected by the organisation IMS Health. We limited the analysis to 189 drugs containing rifampicin, which have fewer non-tuberculosis indications than, for example, fluoroquinolones. These 189 products capture all rifampicin-containing drugs being sold in India between 2013 and 2014. All products were fixed-dose combinations or branded drugs: they were thus sold only in the private sector and not the public sector, which uses different product forms (including loose pills) under non-proprietary names.

**Research in context**

**Evidence before this study**

Not all incident cases of tuberculosis are reported to public health authorities: WHO estimates overall tuberculosis incidence in India by estimating the proportion of incident cases that are notified (the case detection rate [CDR]), and dividing published tuberculosis notifications by this fraction. In 2014, this approach suggested that over 800 000 tuberculosis cases in India escaped diagnosis by the public health-care system: most of these cases are assumed to have been treated in the private sector. However, CDR estimates are based on expert opinion, with the most recent estimate varying substantially from previous years. We searched PubMed for all studies with keywords “India”, “tuberculosis”, “private”, and “burden”, finding 25 studies for articles published in English from inception until May 31, 2016. Most of these related to the quality of tuberculosis care, whereas one study from 2011 assessed the amount of drug sales in the private sector in India and nine other countries. With a focus on market size estimates, this study also presented an illustrative estimate for how many patients were on treatment for tuberculosis.

**Added value of this study**

There is a need for systematic estimates of private sector tuberculosis burden that are independent of expert opinion. We used updated data from 2013 and 2014 for anti-tuberculosis drug sales in the private sector in India, adjusted for indication of use and data capture. With that empirical data, we built on previous work by systematically exploring the effect of assumptions of duration of treatment, and the extent of over-diagnosis of tuberculosis, on the number of patients treated in the private sector. Although there is limited evidence for either of these parameters, we modelled a range of scenarios to assess the feasibility of current estimates based on expert opinion.

**Implications of all the available evidence**

Tuberculosis treatment in the private sector is considerably greater than previous estimates suggest, and estimates of tuberculosis disease burden for India are implausibly low. This study illustrates the need to address the burden of tuberculosis treated by the private sector and improve surveillance. This study also raises an urgent need to revise current estimates of tuberculosis burden, informed by more systematic evidence relating to tuberculosis management in the private sector.

We aimed to estimate the treatment volume, or the total patient-months of treatment for tuberculosis in the private sector, taking account of both the proportion of prescriptions for a given drug that are for tuberculosis, and the proportion of total drug sales that are captured by IMS Health data. We found estimates at the state level in India, as well as on the national level, for 2013 and 2014. We also estimated 95% credible intervals, informed by uncertainty in the input parameters.

### Calculating volume (patient-months) of treatment

Each product is uniquely identified by its product code, indexed *i* in the analysis. We define the following parameters for data in a given state and year: *N_i_* is the IMS data for total packs of product *i* sold, *c_i_* is the proportion of total sales of product *i* that are captured by IMS data, *m_i_* is the total months of tuberculosis treatment represented by one pack of product *i*, and *p_i_* is the proportion of prescriptions containing product *i* that are for tuberculosis. When each of these quantities is specified, the total number of patient-months of treatment (PM), in a given state and year, is then given by a sum over all product codes *i*:

$$\text{PM}=\sum_{i}\frac{N_{i}}{C_{i}}m_{i}p_{i}$$

That is, adjusting sales data (*N_i_*) for IMS data coverage (*c_i_*), the duration of treatment associated with each product form (*m_i_*) and the indications for tuberculosis versus other diseases (*p_i_*). *N_i_* is measured directly. In practice, each of the remaining parameters carries some uncertainty, which we captured by modelling them as random variables, using distributions described below. Using Latin hypercube sampling, we took 10 000 samples for each of the parameters (*c_i_*, *m_i_*, and *p_i_* over all product codes *i*), and then calculated PM for each sample using the equation. From the resulting ensemble of 10 000 estimates for PM, we then obtained the point estimate for the patient-months using the median, and the uncertainty intervals using the 2·5th and 97·5th percentiles. We then repeated this process for each state and year.

### Data sources and probability distributions for input parameters

For the total drug sales *N_i_* we used state-specific data from the IMS Health Drug Sales Audit. These are monthly drug sales data reported to IMS Health by a recruited panel of stockists. We collected monthly drug sales data using invoices raised for sales of goods to retailers and sub-stockists, hospitals and hospital retailers, and dispensing doctors. Overall, IMS Health's combined drug sales audit in the retail, hospital, and dispensing doctors sectors was estimated to account for over 87% of the total Indian pharmaceutical market in 2014.^11^

For the proportion of prescriptions *p_i_* of product *i* that are for tuberculosis, we drew from the IMS Medical Health Audit, consisting of monthly prescription data from a panel of 4600 doctors following internationally recognised medical practice, and translating to over 800 000 prescriptions every month. The panel of doctors is recruited through a sampling exercise that takes into account the region, specialty type, and patient turnover. In these data, if product *i* has *P* prescriptions of which *T* are for tuberculosis, then we modelled *p_i_* as a β-distributed random variable, with shape and scale parameters *T *+ 1 and *P – T *+ 1, respectively. Data are available at the regional level, but not at the state level. Accordingly, for each state we selected the data from the relevant region.

For *m_i_*, we again drew from the Medical Health Audit data. In particular, prescriptions of product code *i* have a certain frequency distribution, available from the audits. Putting this together with the duration of treatment associated with each dose, we constructed the probability distribution for the number of months associated with each prescription of product code *i*. Again, since these data are only regionally stratified, for each state we used the corresponding, region-specific estimates.

Finally for *c_i_*, we used data from IMS Health data validation studies. In brief, at the end of each year, pharmaceutical companies subscribed to IMS are supplied with IMS estimates for their yearly sales volume, for comparison with their actual sales volume. Not all rifampicin-containing products are included in these studies. Accordingly, for each product code in the present work (analysis products), we estimated *c_i_* using those products in the validation study (validation products) having a comparable volume of sales. In particular, we grouped validation products by volume (whether high, moderate, or low volume products), found the mean and variance for each volume category, and then modelled IMS coverage within each category as a normal distribution. By categorising analysis products in the same way, we modelled *c_i_* for each required product using the normal distribution from the relevant volume category.

### Implications of treatment volume for burden (numbers of patients)

Given an estimate of PM in a given year, the corresponding number of patients receiving tuberculosis treatment is given by PM / *D*, where *D* is the average duration (in months) for which patients take tuberculosis treatment in the private sector. However, not all of these patients might genuinely have tuberculosis. To adjust for potential overdiagnosis, we incorporated the positive predictive value (PPV) of tuberculosis diagnosis in the private sector (ie, the proportion of people diagnosed with tuberculosis in the private sector who genuinely have tuberculosis). Therefore, overall the number of patients with tuberculosis receiving private-sector treatment in a given year is estimated simply as PM × (PPV /* D*). In the absence of systematic, quantitative estimates for these parameters, we present results for a range of scenarios for PPV and *D.*

### Patient-months of treatment in the public sector

To compare against the amount of treatment in the public sector, we used RNTCP notifications and, for simplicity, assumed 6 months of treatment for new cases and 9 months of treatment for retreatment. Because some patients might not complete treatment even in the public sector, this approach yields an upper bound for patient-months of treatment. Consequently, this approach would tend to be conservative with respect to the relative amount of treatment in the private versus public sectors (ie, tending to underestimate this quantity).

### Role of the funding source

This work was funded by the Bill & Melinda Gates Foundation. PD is affiliated with the Bill & Melinda Gates Foundation and was involved in the conception of the study, preparation of the manuscript, and interpretation of results, but had no role in the data analysis. The funder otherwise had no role in study design, data collection, data analysis, data interpretation, or writing of the report. The corresponding author had full access to all the data in the study and had final responsibility for the decision to submit for publication.

## Results

Table 1 shows estimates for the total patient-months of treatment (PM) in the private sector in 2013 and 2014, with a comparison against corresponding numbers in the public sector. Overall, estimates are stable between the years, although there is noticeable variation between states in the relative amount of treatment between private and public sectors. At one extreme, Orissa shows the public sector having 1·5–2·8 times as many PM as the private sector (taking the inverse of the ratios shown). At the other extreme, the private sector in Bihar provides over three times as many PM as the public sector. Overall, on a national level in both years, there was roughly twice as much tuberculosis treatment in the private sector as in the public sector. Although the analysis focuses on rifampicin-containing drugs, other tuberculosis drugs (isoniazid and ethambutol) showed similar sales volumes on a national level over this period (appendix).

To translate these population estimates to numbers of patients being treated (whether or not they are genuine tuberculosis cases), table 2 shows estimates for 2014 under different scenarios ranging from 3 months to 9 months, with a comparison against numbers of patients registered for treatment under RNTCP. The appendix shows corresponding estimates for 2013.

Finally, to estimate actual burden of tuberculosis cases in the private sector, the figure shows estimates for 2014, under a range of scenarios for the PPV of tuberculosis diagnosis in the private sector, and for the average duration of treatment in the private sector. For illustration, and in the absence of systematic data on either of these parameters, the diamond marks a moderate set of parameter values: if a patient diagnosed with tuberculosis in the private sector undergoes 4 months of treatment on average, and if 50% of tuberculosis diagnoses in the private sector are genuine cases of tuberculosis, then these figures suggest that 2·2 million genuine cases of tuberculosis were treated in the private sector in 2014 (compared with 1·42 million patients treated in the public sector in the same year). This estimate increases when assuming higher values for PPV, and when assuming shorter average treatment duration.

## Discussion

The vast and fragmented private health-care sector is a prominent feature in the health landscape of India. In the context of tuberculosis, this sector is difficult to study and systematically characterise, yet remains crucial for understanding and managing the overall burden of tuberculosis. In this work, we took advantage of systematic collection of drug sales data in the private sector to address this gap, presenting new estimates that suggest the burden of tuberculosis might be considerably higher than previously recognised.

The key output of this approach is the volume (patient-months) of patient treatment in the private sector, which is twice as much as that provided in the public sector. On any given day, this translates on average to 1·46 million people being on tuberculosis treatment, more than 0·12% of the country's population. Moreover, tuberculosis treatment in the private sector is typically paid for by out-of-pocket expenditure; if a 6-month course of first-line, anti-tuberculosis medication costs US$20, our estimates imply that in 2014, over $59 million was spent in out-of-pocket expenditure on first-line tuberculosis drugs alone.

Estimates by WHO use expert opinion for case detection rates to project from notifications to overall incidence.^9^ The most recent estimates imply that in 2014 about 800 000 patients went untreated by the public sector.^1^ Reconciling these estimates with 17·8 million patient-months of private-sector treatment in 2014 would require either a very low PPV (27% if assuming a 6-month treatment duration) or a long treatment duration (over 11 months if assuming a PPV of 50%). Instead, taking plausible ranges of 40–60% for PPV and 2–6 months for treatment duration suggests that in 2014 alone, 1·19–5·34 million tuberculosis patients received private-sector tuberculosis treatment. The midpoint in this range corresponds to a private-sector tuberculosis burden of 2·2 million cases, more than twice the burden suggested by previous assumptions.

Our findings have implications for the tuberculosis strategy in India. First, the vast disorganised private health-care sector poses major challenges to tuberculosis control. India's RNTCP has committed to providing free, high-quality tuberculosis care to patients in the private sector.^12^ Initiatives such as private-sector engagement to improve tuberculosis care in this sector, offer potential mechanisms for realising these goals.^8^ In this context, our results suggest that the scale of the challenge is substantially larger than has hitherto been appreciated. These findings underscore the need for redoubled efforts to reach patients being treated in the private sector, to deliver the highest possible standards of tuberculosis care.

Second, our work points to the urgent need for further strengthening of tuberculosis surveillance in the private sector. Although there has been increasing notification of tuberculosis cases by the private sector to public health authorities, these accounted in 2014 for 106 414 patients^13^—a level far below that estimated here. Emerging initiatives, such as the proposed provision of free, daily-dosed tuberculosis treatment to all those needing it in the private sector, could bring about important steps in this direction.^14^

Third, our findings highlight uncertainty around the true burden of tuberculosis in India. Methods for estimating this burden should be complemented by independent approaches generating primary data. In addition to the surveillance needs mentioned above, a national prevalence survey would provide direct evidence for the numbers of patients receiving treatment in the private sector. Moreover model-based approaches, such as the Global Burden of Disease study,^15^ offer the capability to collate disparate but important sources for estimating tuberculosis burden. In future, findings such as those presented here could constitute an additional source of evidence for refining these and other analytical approaches.

Previous work on the role of the private sector used interviews of patients diagnosed with tuberculosis in 30 districts in India, to estimate that nearly half of patients were on treatment outside RNTCP.^2^ Relying as it does on self-reported tuberculosis, these estimates can be interpreted as a lower bound of the amount of tuberculosis treatment in the private sector. Another study, also using drug sales in the private sector, cast valuable light on the private market for different tuberculosis drugs.^10^ Our findings for overall treatment volumes in India are broadly consistent. Moreover, Delhi features prominently in our results for the amount of private-sector treatment relative to the public sector (table 1). This result is consistent with other findings in the city,^16^ where engagement with private sector providers led to a greater than ten times increase in tuberculosis notifications, indicative of a large tuberculosis burden being managed by this sector. Although it might be tempting to hold India's large informal health sector responsible for the observed high usage of tuberculosis drugs, recent work from India, using standardised patients, show that anti-tuberculosis drugs are rarely dispensed by pharmacists, informal providers, and practitioners of alternative medical systems.^6^, ^17^ Thus, qualified, allopathic doctors in India are the primary source of anti-tuberculosis drug prescriptions, and should be the target of engagement and antimicrobial stewardship efforts.

Our approach has some limitations. First, we do not have reliable estimates for the PPV of tuberculosis diagnosis in the private sector, nor for the mean duration of treatment (*D*) in the private sector. The estimates that we present for numbers of patients with tuberculosis, under different scenarios, should thus be taken as illustrative, and not definitive. Estimating such parameters in a systematic way is a real challenge. Nonetheless, new methods are emerging, such as the use of standardised patients to assess the quality of tuberculosis care in this sector.^6^ In future, these and other approaches could be valuable in quantifying PPV and *D* more precisely, and more broadly for systematically studying the private health-care sector.

Second, in the simple estimates in the figure, we neglect complexities such as the potential for a patient to receive treatment in the private sector first, and subsequently in the public sector. However, a nationally representative study^18^ in 2010 estimated that such patients accounted for about 8% of all tuberculosis cases that were notified in that year. These findings suggest that the numbers are not so great as to considerably bias our estimates. Further work could aim to extend these findings to more recent years.

Third, there are several types of patient with tuberculosis that the data do not capture. For example, those who could be receiving treatment for tuberculosis in the informal health-care sector, those who have not contacted the health-care system, or those being treated for multidrug-resistant tuberculosis in the private sector. Moreover, there is evidence to suggest that some patients could be treated for tuberculosis with other drugs such as fluoroquinolones in the private sector,^6^ although there is no systematic evidence for the proportion of patients receiving these drugs in combination with rifampicin-containing products. Nonetheless, taken together, all these factors would suggest that the true burden of tuberculosis is even greater than suggested in the present analysis.

Overall, the approach described here cannot replace traditional approaches to surveillance, including routine notifications and periodic surveys. There remains a pressing need to strengthen and widen these systems. Nonetheless, the implications of this analysis could offer additional perspectives on such a vast and complex health-care system as in India. In future these and other approaches, in combination with existing and improved sources of data, could help to build a truly comprehensive picture of the burden of tuberculosis in India.

## Figures and Tables

**Figure:**
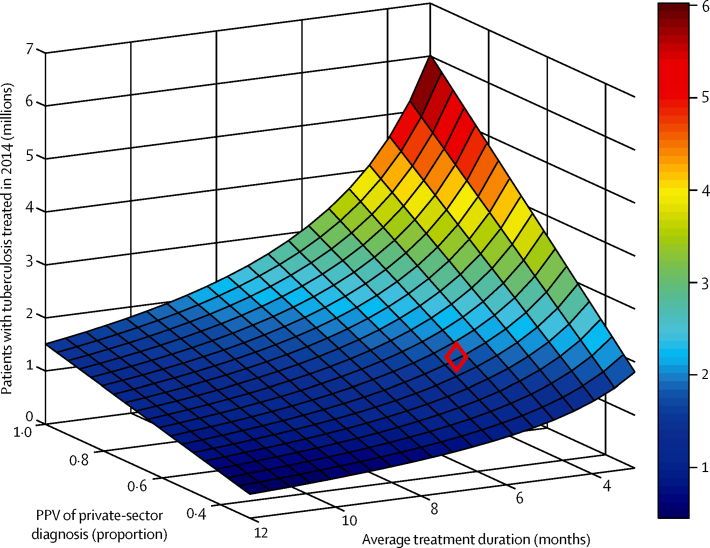
Implications of treatment volume in 2014 for tuberculosis burden managed by the private sector in India Estimates are shown for the number of patients with tuberculosis treated by the private sector (see colour bar for numbers in millions), under different scenarios for the average duration of treatment in the private sector, and the proportion of private-sector tuberculosis diagnoses that genuinely have tuberculosis. The diamond illustrates a moderate parameter regime, in which 50% of diagnoses in the private sector genuinely have tuberculosis, and the average treatment duration is for 4 months. This corresponds to an estimated 2·2 million patients being treated in the private sector in 2014.

**Table 1:** Patient-months of treatment in 2013 and 2014 across India

	**Patient-months in 2013**			**Patient-months in 2014**		
	Private sector (thousands)	Public sector (thousands)	Ratio, private to public	Private sector (thousands)	Public sector (thousands)	Ratio, private to public
Andhra Pradesh	1020 (793–1328)	683	1·5 (1·2–1·9)	947 (736–1258)	709	1·3 (1·0–1·8)
Assam & North East	344 (277–458)	348	1·0 (0·8–1·3)	375 (298–518)	364	1·0 (0·8–1·4)
Bihar	1560 (1357–1892)	441	3·5 (3·1–4·3)	1567 (1356–1950)	446	3·5 (3·0–4·4)
Chhattisgarh	300 (244–380)	163	1·8 (1·5–2·3)	266 (218–341)	183	1·5 (1·2–1·9)
Delhi	1175 (934–1504)	335	3·5 (2·8–4·5)	1108 (880–1496)	356	3·1 (2·5–4·2)
Goa	18 (14–26)	11	1·6 (1·2–2·3)	19 (13–28)	10	1·8 (1·3–2·7)
Gujarat	1044 (837–1292)	501	2·1 (1·7–2·6)	976 (790–1231)	525	1·9 (1·5–2·3)
Haryana	357 (290–452)	258	1·4 (1·1–1·8)	353 (286–459)	266	1·3 (1·1–1·7)
Himachal Pradesh	48 (38–67)	90	0·5 (0·4–0·7)	54 (41–78)	95	0·6 (0·4–0·8)
Jammu & Kashmir	180 (145–240)	72	2·5 (2·0–3·3)	133 (108–180)	68	2·0 (1·6–2·7)
Jharkhand	309 (265–392)	225	1·4 (1·2–1·7)	377 (314–495)	231	1·6 (1·4–2·1)
Karnataka	556 (409–744)	406	1·4 (1–1·8)	558 (395–776)	404	1·4 (1·0–1·9)
Kerala	220 (168–293)	154	1·4 (1·1–1·9)	174 (133–237)	149	1·2 (0·9–1·6)
Madhya Pradesh	1166 (985–1413)	599	1·9 (1·6–2·4)	1008 (837–1241)	644	1·6 (1·3–1·9)
Maharashtra	1639 (1296–2074)	906	1·8 (1·4–2·3)	1623 (1257–2063)	890	1·8 (1·4–2·3)
Orissa	123 (103–171)	288	0·4 (0·4–0·6)	141 (115–188)	292	0·5 (0·4–0·6)
Punjab	461 (386–587)	265	1·7 (1·5–2·2)	403 (329–521)	268	1·5 (1·2–1·9)
Rajasthan	1063 (900–1307)	595	1·8 (1·5–2·2)	1039 (865–1274)	596	1·7 (1·5–2·1)
Tamilnadu	672 (508–891)	530	1·3 (1·0–1·7)	619 (467–825)	559	1·1 (0·8–1·5)
Uttar Pradesh	4942 (4214–6232)	1615	3·1 (2·6–3·9)	5041 (4292–6601)	1600	3·2 (2·7–4·1)
Uttaranchal	328 (278–445)	85	3·8 (3·3–5·2)	331 (275–431)	96	3·4 (2·9–4·5)
West Bengal	390 (317–552)	602	0·6 (0·5–0·9)	470 (373–680)	578	0·8 (0·6–1·2)
National	18 118 (16 993–19 717)	9180	2·0 (1·9–2·1)	17 793 (16 709–19 841)	9340	1·9 (1·8–2·1)

Data in parentheses are 95% credible intervals. Private sector represents estimates from IMS data. Public sector numbers are obtained using Revised National TB Control Programme notifications and assuming treatment durations of 6 months and 9 months for new and retreatment cases, respectively. For conciseness, the smallest states have been aggregated as follows: North East includes Arunachal Pradesh, Manipur, Meghalaya, Mizoram, Nagaland, and Tripura; Gujarat includes Gujarat and Daman & Diu; Kerala includes Kerala and Lakshadweep; Maharashtra includes Maharashtra and Dadar and Nagar Haveli; Punjab includes Punjab and Chandigarh; Tamil Nadu includes Tamil Nadu, Pondicherry, and Andaman & Nicobar; West Bengal includes West Bengal and Sikkim.

**Table 2:** Estimated numbers of patients receiving tuberculosis treatment in 2014 across India

	**Patients in private sector (thousands)**			**Patients in public sector (thousands)**
	3 month duration	6 month duration	9 month duration	
Andhra Pradesh	315 (245–419)	157 (122–209)	105 (81–139)	107
Assam	125 (99–172)	62 (49–86)	41 (33–57)	55
Bihar	522 (452–650)	261 (226–325)	174 (150–216)	67
Chhattisgarh	88 (72–113)	44 (36–56)	29 (24–37)	28
Delhi	369 (293–498)	184 (146–249)	123 (97–166)	53
Goa	6 (4–9)	3 (2–4)	2 (1–3)	1
Gujarat	325 (263–410)	162 (131–205)	108 (87–136)	77
Haryana	117 (95–153)	58 (47–76)	39 (31–51)	39
Himachal Pradesh	18 (13–26)	9 (6–13)	6 (4–8)	14
Jammu & Kashmir	44 (36–60)	22 (18–30)	14 (12–20)	10
Jharkhand	125 (104–165)	62 (52–82)	41 (34–55)	35
Karnataka	186 (131–258)	93 (65–129)	62 (43–86)	61
Kerala	58 (44–79)	29 (22–39)	19 (14–26)	23
Madhya Pradesh	336 (279–413)	168 (139–206)	112 (93–137)	99
Maharashtra	541 (419–687)	270 (209–343)	180 (139–229)	133
Orissa	47 (38–62)	23 (19–31)	15 (12–20)	45
Punjab	134 (109–173)	67 (54–86)	44 (36–57)	40
Rajasthan	346 (288–424)	173 (144–212)	115 (96–141)	90
Tamilnadu	206 (155–275)	103 (77–137)	68 (51–91)	85
Uttar Pradesh	1680 (1430–2200)	840 (715–1100)	560 (476–733)	245
Uttaranchal	110 (91–143)	55 (45–71)	36 (30–47)	14
West Bengal	156 (124–226)	78 (62–113)	52 (41–75)	88
National	5931 (5569–6613)	2965 (2784–3306)	1977 (1856–2204)	1421

Data in parentheses are 95% credible intervals. In the private sector, estimates are shown under different assumptions for the average duration of treatment, ranging from 3 months to 9 months. In the public sector, the total number of cases registered for treatment by the Revised National TB Control Programme in 2014 are shown. In the private sector, not all patients receiving tuberculosis treatment might genuinely have tuberculosis: the figures are adjusted for potential overdiagnosis in the private sector.
