# Supplementary material for: The number of privately treated tuberculosis cases in India: an estimation from drug sales data
Source: Lancet Infect Dis. 2016 Nov;16(11):1255–60. doi: 10.1016/S1473-3099(16)30259-6 (PMC5067370; doi:10.1016/S1473-3099(16)30259-6)
Supplement: Supplementary appendix [file mmc1.pdf]

THE LANCET Infectious Diseases

Supplementary webappendix

This webappendix formed part of the original submission and has been peer reviewed. We post it as supplied by the authors.

Supplement to: Arinaminpathy N, Batra D, Khaparde S, et al. The number of privately treated tuberculosis cases in India: an estimation from drug sales data. *Lancet Infect Dis* 2016; published online Aug 24. http://dx.doi.org/10.1016/S1473-3099(16)30259-6.

How many tuberculosis cases are treated outside the public health sector in India? An approach using private-sector drug sales data

Appendix

Sample data tables

The analysis involved 189 different product codes over 22 regions in India, involving the sale of 141.8 million packs of rifampicin-containing medicine in 2013 and 143.3 million packs in 2014.

Below we give illustrative tables for the data inputs; for demonstration, we take data relevant to Andhra Pradesh in 2013, concentrating on the ten product codes accounting for the most numbers of packs sold.

Table S1 shows the total sales of each of these product codes, along with the product details (product name and number of units in a pack). The column 'Total packs sold' provides the data for N_i in equation (1) in the main text.

Product code	Pack	Pack Size	State	Total packs sold	Volume category
**130005**	AKT-4 COMBIPACK x 4	4	ANDHRA PRADESH	1979744	High
**129505**	AKT-3 COMBIPACK x 2	2	ANDHRA PRADESH	1274474	High
**2712707**	FORECOX FILM C.TABS x 6	6	ANDHRA PRADESH	942463	High
**5244025**	R-CINEX TABS 600 MG x 3 (/300)	3	ANDHRA PRADESH	557939	High
**5244004**	R-CINEX CAPS 450 MG x 10 (/300)	10	ANDHRA PRADESH	420627	High
**4101605**	MONTO-4 CAPLET KIT x 2	2	ANDHRA PRADESH	277348	High
**2714405**	FORECOX KIT TAB-CAP.KIT x 4	4	ANDHRA PRADESH	245715	High
**130410**	AKURIT-4 FILM C.TABS x 10	10	ANDHRA PRADESH	210891	High
**2713105**	FORECOX-150 FILM C.TABS x 6	6	ANDHRA PRADESH	190974	High
**5372610**	RHE-KIT TABS KIT x 2	2	ANDHRA PRADESH	140087	High

Table S1: Example data for total sales of rifampicin-containing drugs in Andhra Pradesh in 2013. Shown are the 10 drugs accounting for the most numbers of packs sold (representing 72% of the total in Andhra Pradesh in 2013). For demonstration, the table also shows pack names and sizes: these are dropped in subsequent tables and in the analysis, using only the product code as a unique identifier.

To estimate p_i in equation (1), the proportion of sales of product *i* that are for TB, Table S2 shows information for the numerator and denominator. As described in the main text, these terms are used to construct a beta distribution for p_i for each product. This information, available from IMS Health prescription audits, is available at the regional level (here, showing data for the 'South' region where Andhra Pradesh belongs).

Product code	Region	Numerator for p_i	Denominator for p_i
**130005**	South	12201	14667
**129505**	South	6561	7840
**2712707**	South	219	250
**5244025**	South	5340	6146
**5244004**	South	5583	6910
**4101605**	South	909	1076
**2714405**	South	70	70
**130410**	South	2663	3223
**2713105**	South	554	830
**5372610**	South	30	44

Table S2: Data for the numbers of prescriptions involving a given product code, as captured by the IMS Health prescription audit survey. In the column headings, the 'numerator' is the total number of prescriptions that mentioned TB as a diagnosis, and the 'denominator' is the total number of prescriptions with any stated diagnosis.

To translate packs sold to patient-days of treatment per pack (d_i), we drew again from the prescription audits. Taking the example of the top-selling pack code 130005, Table S3 shows the frequency distribution for prescribed days of treatment per prescription, in the South region.

Treatment duration (days)	2/3	0.8	1	4/3	2	4	8	28
**Relative frequency (%)**	1.32	0.28	5.40	2.00	3.81	86.02	0.93	0.25

Table S3: Frequency distribution for the duration of treatment represented by each prescription of product code 130005 in the 'South' region, as captured by the IMS Health prescription audit survey. As stated in table S2, these proportions relate to a total of 12201 TB prescriptions. The product code illustrated here represents an "AKT-4 combipack" supplied as four strips (see table S1), with each strip providing a single day of treatment: a full treatment course (several months of daily treatment) thus may involve a series of prescriptions for this product.

Finally, estimates for c_i are available only for a limited number of rifampicin-containing products. As described in the main text, to estimate c_i for the remaining required products, we matched by category of sales volumes. With the column 'volume category' in Table S1 showing categories for each rifampicin-containing product, the parameters in Table S4 show the distributions for c_i associated with each category.

Volume category	Mean	Standard deviation
**Low-volume**	1.04	0.18
**Medium-volume**	1.04	0.06
**High-volume**	1.03	0.09

Table S4: Parameters of a normal distribution used to capture potential over- or under-estimates in IMS Health sales data, as captured by IMS Health validation studies.

For comparison with other TB drugs, Table S5 summarizes aggregate sales data in 2014 for rifampicin, and two other drugs used to treat TB. As above, the proportion of prescriptions that are for TB is drawn from prescription audits for each of these drugs. Overall, the data illustrates that national sales volumes for different TB drugs have a scale consistent with those of rifampicin.

Drugs containing:	Total units sold in 2014 (million)	Aevrage proportion prescribed for TB (%)
**Rifampicin**	143.3	86
**Isoniazid**	140.0	85
**Ethambutol**	110.8	89

Table S5. Summary sales and usage data for products containing three different types of TB drugs, for comparison with rifampicin. 'Total units' denotes the sum total of drugs sold in both fixed-dose combination (FDC) and plain dosage forms. Note that Isoniazid and Ehtambutol are typically not prescribed alone, but in combination with Rifampicin.

State	Patients in private sector (thousands)			Patients in public sector (thousands)
	3 month duration	6 month duration	9 month duration	
**ANDHRA PRADESH**	340 (264 - 442)	170 (132 - 221)	113 (88 - 147)	103
**ASSAM**	114 (92 - 152)	57 (46 - 76)	38 (30 - 50)	53
**BIHAR**	520 (452 - 630)	260 (226 - 315)	173 (150 - 210)	66
**CHHATTISGARH**	100 (81 - 126)	50 (40 - 63)	33 (27 - 42)	25
**DELHI**	391 (311 - 501)	195 (155 - 250)	130 (103 - 167)	50
**GOA**	6 (4 - 8)	3 (2 - 4)	2 (1 - 2)	1
**GUJARAT**	348 (279 - 430)	174 (139 - 215)	116 (93 - 143)	73
**HARYANA**	119 (96 - 150)	59 (48 - 75)	39 (32 - 50)	38
**HIMACHAL PRADESH**	16 (12 - 22)	8 (6 - 11)	5 (4 - 7)	13
**JAMMU & KASHMIR**	60 (48 - 80)	30 (24 - 40)	20 (16 - 26)	11
**JHARKHAND**	103 (88 - 130)	51 (44 - 65)	34 (29 - 43)	34
**KARNATAKA**	185 (136 - 248)	92 (68 - 124)	61 (45 - 82)	61
**KERALA**	73 (56 - 97)	36 (28 - 48)	24 (18 - 32)	24
**MADHYA PRADESH**	388 (328 - 471)	194 (164 - 235)	129 (109 - 157)	92
**MAHARASHTRA**	546 (432 - 691)	273 (216 - 345)	182 (144 - 230)	135
**ORISSA**	41 (34 - 57)	20 (17 - 28)	13 (11 - 19)	45
**PUNJAB**	153 (128 - 195)	76 (64 - 97)	51 (42 - 65)	39
**RAJASTHAN**	354 (300 - 435)	177 (150 - 217)	118 (100 - 145)	90
**TAMIL NADU**	224 (169 - 297)	112 (84 - 148)	74 (56 - 99)	81
**UTTAR PRADESH**	1647 (1404 - 2077)	823 (702 - 1038)	549 (468 - 692)	247
**UTTARANCHAL**	109 (92 - 148)	54 (46 - 74)	36 (30 - 49)	12
**WEST BENGAL**	130 (105 - 184)	65 (52 - 92)	43 (35 - 61)	91
**National**	6039 (5664 - 6572)	3019 (2832 - 3286)	2013 (1888 - 2190)	1395

Table S6. Estimated numbers of patients receiving TB treatment in 2013. Columns are as in table 2 in the main text, but showing corresponding estimates for 2013.
